# Supplementary material for: The lawful imprecision of human surface tilt estimation in natural scenes
Source: eLife. 2018 Jan 31;7:e31448. doi: 10.7554/eLife.31448 (PMC5844693; doi:10.7554/eLife.31448)
Supplement: Transparent reporting form [file elife-31448-transrepform.pdf]

eLIFE
1st Floor
24 Hills Road
Cambridge CB2 1JP, UK
P 01223 855340
W elifesciences.org
T @elife

eLife's transparent reporting form

We encourage authors to provide detailed information *within their submission* to facilitate the interpretation and replication of experiments. Authors can upload supporting documentation to indicate the use of appropriate reporting guidelines for health-related research (see EQUATOR Network), life science research (see the BioSharing Information Resource), or the ARRIVE guidelines for reporting work involving animal research. Where applicable, authors should refer to any relevant reporting standards documents in this form.

If you have any questions, please consult our Journal Policies and/or contact us: editorial@elifesciences.org.

Sample-size estimation

- You should state whether an appropriate sample size was computed when the study was being designed
- You should state the statistical method of sample size computation and any required assumptions
- If no explicit power analysis was used, you should describe how you decided what sample (replicate) size (number) to use

Please outline where this information can be found within the submission (e.g., sections or figure legends), or explain why this information doesn't apply to your submission:

Confidence intervals were computed for the results presented in the manuscript via bootstrapping or via Monte Carlo simulation, as appropriate. In many cases, the confidence intervals were smaller than the data points and thus were not depicted in the figures.

Replicates

- You should report how often each experiment was performed
- You should include a definition of biological versus technical replication
- The data obtained should be provided and sufficient information should be provided to indicate the number of independent biological and/or technical replicates
- If you encountered any outliers, you should describe how these were handled
- Criteria for exclusion/inclusion of data should be clearly stated
- High-throughput sequence data should be uploaded before submission, with a private link for reviewers provided (these are available from both GEO and ArrayExpress)

Please outline where this information can be found within the submission (e.g., sections or figure legends), or explain why this information doesn't apply to your submission:

No data was excluded from the analyses. Data was collected from Subject 1 over the course of 4 days. Data was collected from Subject 2 over the course of 2 weeks. Data was collected from Subject 3 over the course of 3 weeks.

eLife Sciences Publications, Ltd is a limited liability non-profit non-stock corporation incorporated in the State of Delaware, USA, with company number 5030732, and is registered in the UK with company number FC030576 and branch number BR015634 at the address 1st Floor, 24 Hills Road, Cambridge CB2 1JP | August 2014

Statistical reporting

- Statistical analysis methods should be described and justified
- Raw data should be presented in figures whenever informative to do so (typically when N per group is less than 10)
- For each experiment, you should identify the statistical tests used, exact values of N, definitions of center, methods of multiple test correction, and dispersion and precision measures (e.g., mean, median, SD, SEM, confidence intervals; and, for the major substantive results, a measure of effect size (e.g., Pearson's r, Cohen's d)
- Report exact p-values wherever possible alongside the summary statistics and 95% confidence intervals. These should be reported for all key questions and not only when the p-value is less than 0.05.

Please outline where this information can be found within the submission (e.g., sections or figure legends), or explain why this information doesn't apply to your submission:

1. Raw data is presented in Figure 2C.

2. Circular mean (Figure 2E) and variance (Figure 2F) are computed from 300 samples (for natural stimuli) and 120 samples (for artificial stimuli) in each bin. Definitions of circular mean and variance are provided in Methods (Data analysis).

3. To assess the similarity between human and model errors, which are angular variables, we computed the circular correlation coefficients

$$\rho = \frac{\sum_i \sin(\alpha_i - \bar{\alpha})\sin(\beta_i - \bar{\beta})}{\sqrt{\sum_i \sin^2(\alpha_i - \bar{\alpha})\sin^2(\beta_i - \bar{\beta})}}$$

where α and β are the samples of angular data, and $\bar{\alpha}$ and $\bar{\beta}$ are the circular means (Figure 6). 95% confidence intervals, computed from 1000 bootstrapped datasets, are shown in Figure 6B,D.

(For large datasets, or papers with a very large number of statistical tests, you may upload a single table file with tests, Ns, etc., with reference to sections in the manuscript.)

Group allocation

- Indicate how samples were allocated into experimental groups (in the case of clinical studies, please specify allocation to treatment method); if randomization was used, please also state if restricted randomization was applied
- Indicate if masking was used during group allocation, data collection and/or data analysis

Please outline where this information can be found within the submission (e.g., sections or figure legends), or explain why this information doesn't apply to your submission:

N/A

Additional data files ("source data")

- We encourage you to upload relevant additional data files, such as numerical data that are represented as a graph in a figure, or as a summary table

- Where provided, these should be in the most useful format, and they can be uploaded as "Source data" files linked to a main figure or table
- Include model definition files including the full list of parameters used
- Include code used for data analysis (e.g., R, MatLab)
- Avoid stating that data files are "available upon request"

Please indicate the figures or tables for which source data files have been provided:

Two source data files are included: (1) human.txt for tilt estimates from three human subjects for natural and artificial stimuli experiments; and (2) mmse.txt for tilt estimates from the optimal model for the natural stimuli. They are tab-separated values files.

1. human.txt file contains the columns:
stmType: NAT (natural stimuli) or ART (natural stimuli)
subjNum: subject number
winSize: window diameter size in degrees of visual angle 1 (small) or 3 (large)
trialID: trial number
PTiltSDeg: groundtruth signed tilt
PSlantDeg: groundtruth slant
PDist: groundtruth distance
PTiltVarU: tilt contrast power
RTiltSDeg: signed tilt estimates

2. mmse.txt file contains the columns:
stmType: NAT (natural stimuli) or ART (natural stimuli)
subjNum: subject number
trialID: trial number
RTiltUDeg: unsigned tilt estimates

3. human_fullfieldnatural.txt contains the columns that are the same with human.txt except winSize.

3. The data represent the graphs in
Figure 2C-F, supplement 1, 2, 3B-D, 4A, 4B, 5
Figure 3
Figure 4, supplement 1
Figure 5C, supplement 1
Figure 6A,C, supplement 1 A,C
Figure 7
Figure 8A,C, supplement 1
Figure 9, supplement 1

eLife Sciences Publications, Ltd is a limited liability non-profit non-stock corporation incorporated in the State of Delaware, USA, with company number 5030732, and is registered in the UK with company number FC030576 and branch number BR015634 at the address 1st Floor, 24 Hills Road, Cambridge CB2 1JP | August 2014
